# Laparoscopic reduction of strangulated small bowel incarceration by controlled enlargement of a uterine perforation following manual vacuum aspiration

**DOI:** 10.1093/jscr/rjag1018

**Published:** 2026-11-04

**Authors:** Miki Ikeda, Satomi Kan, Yumiko Takeuchi, Yuri Takemura, Hiroshi Sato, Yuko Imamura, Kazuyo Kakui

**Affiliations:** Department of Obstetrics and Gynecology, Hyogo Prefectural Amagasaki General Medical Center, 2-17-77 Higashinaniwa, Amagasaki, Hyogo, 660-8550, Japan; Department of Obstetrics and Gynecology, Hyogo Prefectural Amagasaki General Medical Center, 2-17-77 Higashinaniwa, Amagasaki, Hyogo, 660-8550, Japan; Department of Obstetrics and Gynecology, Hyogo Prefectural Amagasaki General Medical Center, 2-17-77 Higashinaniwa, Amagasaki, Hyogo, 660-8550, Japan; Department of Obstetrics and Gynecology, Hyogo Prefectural Amagasaki General Medical Center, 2-17-77 Higashinaniwa, Amagasaki, Hyogo, 660-8550, Japan; Department of Obstetrics and Gynecology, Hyogo Prefectural Amagasaki General Medical Center, 2-17-77 Higashinaniwa, Amagasaki, Hyogo, 660-8550, Japan; Department of Obstetrics and Gynecology, Hyogo Prefectural Amagasaki General Medical Center, 2-17-77 Higashinaniwa, Amagasaki, Hyogo, 660-8550, Japan; Department of Obstetrics and Gynecology, Hyogo Prefectural Amagasaki General Medical Center, 2-17-77 Higashinaniwa, Amagasaki, Hyogo, 660-8550, Japan

**Keywords:** bowel incarceration, manual vacuum aspiration, uterine perforation, laparoscopy, computed tomography, early pregnancy loss

## Abstract

Manual vacuum aspiration (MVA) is widely used for early pregnancy loss, but uterine perforation may cause serious intra-abdominal complications. Bowel incarceration through a uterine perforation can be difficult to recognize because clinical manifestations may be nonspecific. A 37-year-old woman underwent MVA for missed miscarriage at 6 weeks of gestation. After unsuccessful repeat suction for presumed intrauterine hematoma, she developed abdominal pain and vomiting. Contrast-enhanced computed tomography (CT) revealed small bowel herniation through a posterior uterine wall defect with reduced enhancement, suggesting strangulation. Emergency laparoscopy confirmed bowel incarceration. Controlled enlargement of the uterine defect enabled atraumatic bowel reduction; ischemic intestine was resected, and the uterus was repaired laparoscopically. This case demonstrates that bowel incarceration may present with mild findings despite ischemia and highlights the importance of prompt CT and avoidance of repeated intrauterine instrumentation when uterine perforation is suspected.

## Introduction

Manual vacuum aspiration (MVA) is a safe and effective surgical treatment for early pregnancy loss [1]. Uterine perforation is an uncommon complication of intrauterine instrumentation [2], and small bowel incarceration through the defect may cause obstruction, strangulation, and ischemic necrosis [3, 4]. Because clinical and ultrasonographic findings may be nonspecific, diagnosis can be delayed [3, 4]. We report a case successfully managed by laparoscopic controlled enlargement of the uterine defect, bowel reduction, and uterine repair.

## Case report

A 37-year-old woman (gravida 2, para 1) conceived through in vitro fertilization and was diagnosed with missed miscarriage at 6 weeks of gestation. She underwent MVA at a local clinic. During the procedure, resistance was encountered while inserting the cannula, and the device repeatedly caught against the uterine wall. Abnormal passage of the cannula raised concern for uterine perforation. However, her vital signs remained stable and no signs of intraperitoneal bleeding were detected. She was discharged with follow-up instructions. The following day, transvaginal ultrasonography suggested intrauterine hematoma, and repeat suction was attempted but was unsuccessful. Several hours later, she developed progressive abdominal pain and vomiting and was referred to our hospital with suspected uterine perforation. On admission, she was hemodynamically stable. Physical examination revealed mild lower abdominal tenderness without peritoneal signs. Laboratory tests demonstrated mild inflammatory marker elevation. Transvaginal ultrasonography showed a 53-mm hypoechoic intrauterine mass and a small amount of fluid in the vesicouterine pouch (Fig. 1). These findings were initially interpreted as hematoma.

**Figure 1:**
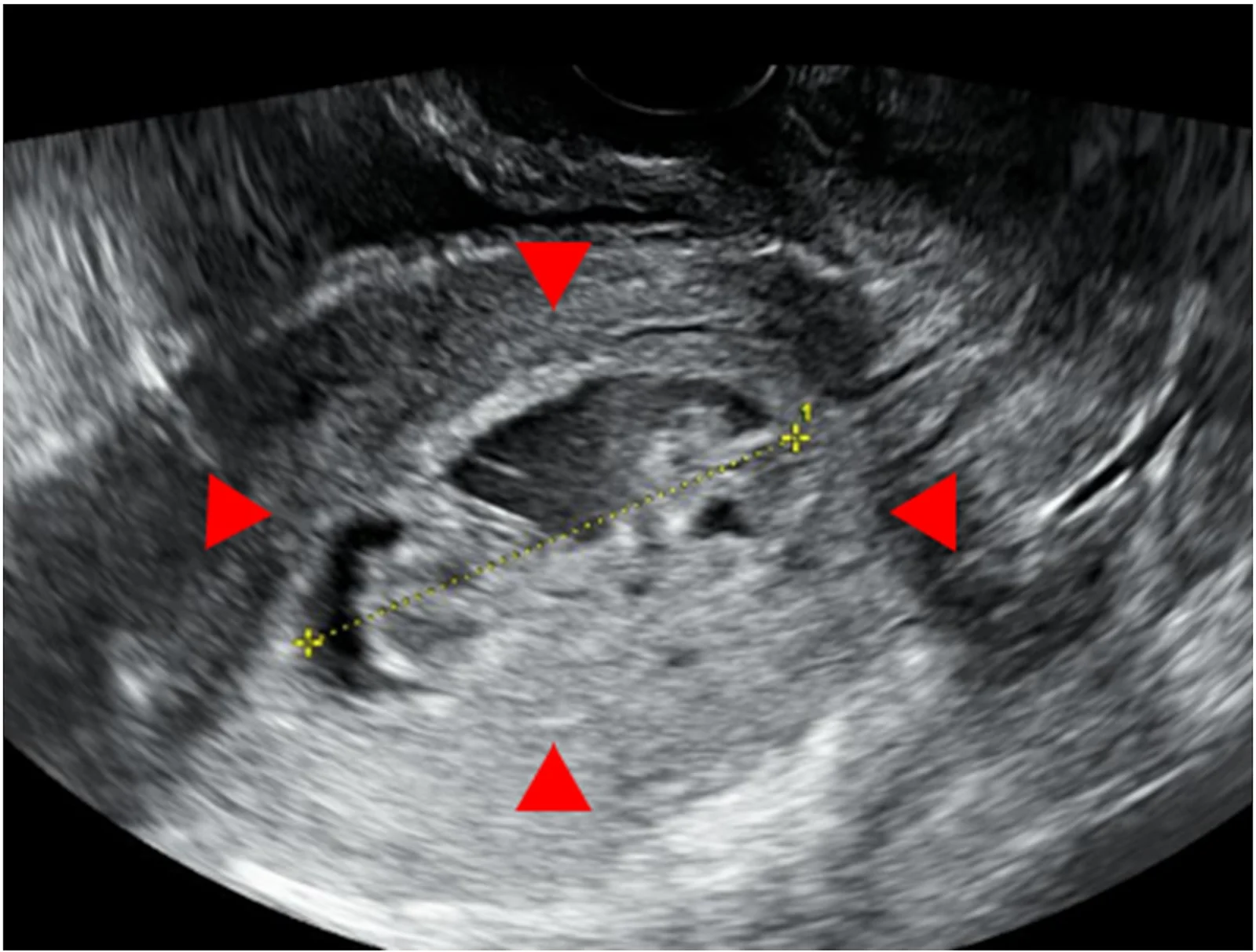
Transvaginal ultrasonography showing a hypoechoic intrauterine mass (arrowhead), initially interpreted as an intrauterine hematoma.

Contrast-enhanced computed tomography (CT) revealed a 1.5-cm posterior uterine wall defect with small bowel herniation into the uterine cavity. The incarcerated bowel demonstrated reduced contrast enhancement compared with adjacent bowel loops, raising concern for strangulated bowel with ischemia (Fig. 2A and B). Emergency laparoscopy with the gastrointestinal surgery team confirmed a 1.5-cm posterior uterine wall perforation. A loop of small intestine was tightly incarcerated through the defect into the uterine cavity (Fig. 3A). Because gentle traction alone was insufficient for safe reduction, the uterine defect was carefully extended laparoscopically by ~1 cm using cold scissors (Fig. 3B). This allowed atraumatic reduction of the incarcerated bowel. The involved segment appeared dark and nonviable, indicating ischemic necrosis (Fig. 3C). Approximately 20 cm of small intestine was resected via mini-laparotomy and functional end-to-end anastomosis was performed. The uterine defect was subsequently repaired laparoscopically with interrupted absorbable sutures in two layers to restore the integrity of the uterine wall (Fig. 3D). Operative time was 195 min and blood loss was minimal. The postoperative course was uneventful, and the patient was discharged on postoperative Day 9. Before discharge, transvaginal ultrasonography showed no thinning or hematoma at the repaired uterine wall, although a vascular intrauterine mass was identified near the fundus. Contrast-enhanced magnetic resonance imaging (MRI) subsequently suggested retained products of conception, which were managed expectantly. The lesion had completely resolved and the serum human chorionic gonadotropin level had normalized by postoperative Day 154. MRI obtained on postoperative Day 37 showed no apparent myometrial thinning at the repaired perforation site compared with the initial CT findings. The patient remained asymptomatic throughout follow-up. Subsequently, the patient experienced one first-trimester miscarriage managed by ultrasound-guided MVA at our institution without complication. She later conceived again through in vitro fertilization and, at the time of manuscript preparation, the pregnancy had progressed uneventfully to ~25 weeks of gestation under the care of another hospital.

**Figure 2:**
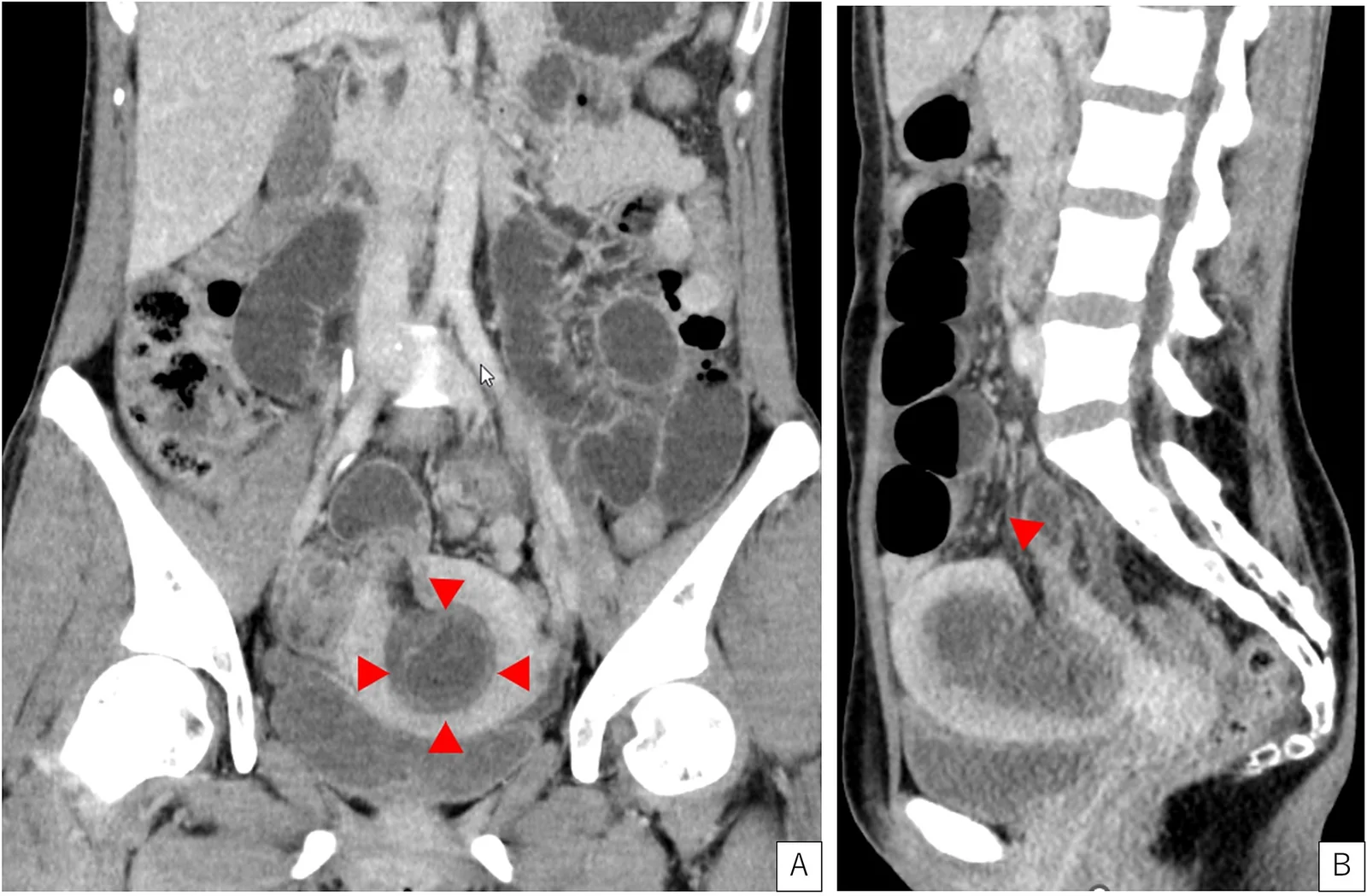
Contrast-enhanced CT demonstrating a defect in the posterior uterine wall with herniation of the small bowel into the uterine cavity (arrowhead). Reduced bowel wall enhancement suggested strangulation and ischemia.

**Figure 3:**
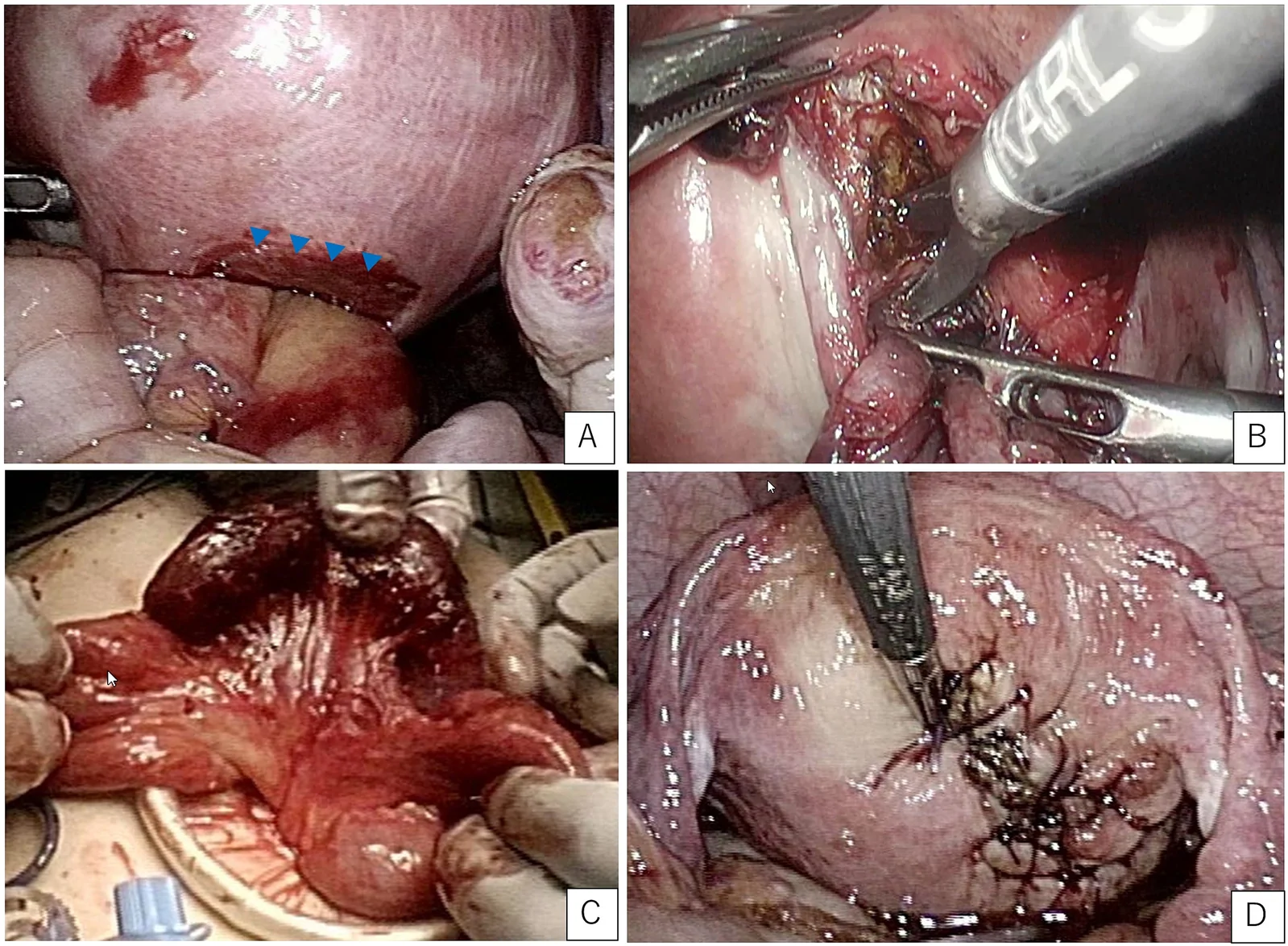
Intraoperative laparoscopic findings. (A) Small bowel tightly incarcerated through the posterior uterine perforation. (B) Controlled enlargement of the uterine defect using cold scissors while the incarcerated bowel was gently displaced with forceps to avoid bowel injury. (C) Ischemic nonviable bowel after reduction. (D) Two-layer laparoscopic repair of the uterine defect.

## Discussion

Risk factors for uterine perforation include gestational age, uterine position, previous uterine surgery, cervical stenosis, and operator-related factors [2, 5]. Abnormal cannula passage, loss of resistance, or unsuccessful aspiration should prompt evaluation for perforation and visceral injury.

Small bowel incarceration through a uterine perforation is an uncommon but potentially life-threatening complication. A systematic review identified four mechanisms of small bowel obstruction after uterine perforation, with substantial variation in both clinical presentation and the interval to diagnosis [3]. Patients may initially exhibit only mild or nonspecific symptoms, and delayed presentation from several weeks to months after the causative procedure has been reported [4, 6, 7]. In the present case, the patient remained hemodynamically stable and exhibited only mild abdominal findings despite strangulation and ischemic necrosis of the incarcerated bowel.

Ultrasonography may misidentify incarcerated bowel as intrauterine hematoma or retained products of conception. Reported findings include intrauterine cystic structures, a multilayered bowel wall, peristalsis, and Doppler vascularity [7, 8]. Nevertheless, diagnosis may remain difficult. When perforation is suspected after unsuccessful aspiration or persistent symptoms, contrast-enhanced CT should not be delayed because it can identify bowel herniation and signs of ischemia [9].

Surgical management should be individualized according to the patient’s hemodynamic status, extent of visceral injury, and bowel viability. Sebai *et al.* recently reported small bowel incarceration after second-trimester abortion diagnosed by CT; the incarcerated bowel remained viable and was reduced surgically, followed by repair of the uterine defect [10]. In contrast, our patient had tightly incarcerated ischemic bowel that could not be safely reduced by gentle traction. Laparoscopic controlled enlargement of the uterine defect using cold scissors enabled atraumatic reduction while preserving the surrounding myometrium. Laparoscopy also permitted assessment of bowel viability and definitive uterine repair, although segmental bowel resection through a mini-laparotomy was required because of ischemic necrosis.

Follow-up MRI showed no myometrial thinning at the repaired uterine wall. Subsequent uncomplicated intrauterine instrumentation and an ongoing pregnancy suggest preserved uterine integrity, although long-term obstetric outcomes remain uncertain.

When uterine perforation is suspected, repeat intrauterine instrumentation should be avoided until bowel injury has been excluded. This case demonstrates that strangulated bowel incarceration may occur despite mild clinical findings and highlights the roles of early contrast-enhanced CT and minimally invasive surgical management.
